# Macroscale abundance patterns of hydromedusae in the temperate Southwestern Atlantic (27°–56° S)

**DOI:** 10.1371/journal.pone.0217628

**Published:** 2019-06-19

**Authors:** María Sofía Dutto, Carlo Javier Chazarreta, Carolina Soledad Rodriguez, Agustín Schiariti, Luciana Mabel Diaz Briz, Gabriel Néstor Genzano

**Affiliations:** 1 Instituto Argentino de Oceanografía (IADO, CONICET-UNS), Centro Científico Tecnológico Bahía Blanca, Bahía Blanca, Argentina; 2 Instituto de Investigaciones Marinas y Costeras (IIMyC, CONICET-UNMdP), Mar del Plata, Argentina; 3 Instituto Nacional de Investigación y Desarrollo Pesquero (INIDEP), Mar del Plata, Argentina; 4 Departamento de Ciencias Marinas, Facultad de Ciencias Exactas y Naturales, Universidad Nacional de Mar del Plata (UNMdP), Mar del Plata, Argentina; University of Sydney, AUSTRALIA

## Abstract

Gelatinous organisms are crucial components of marine ecosystems and some species imply social and economic consequences. However, certain geographic areas, such as the temperate Southwestern Atlantic (SWA, 27° - 56° S), remain understudied in terms of jellyfish ecological data. We analyzed 3,727 plankton samples collected along ~6.7 million km^2^ over a 31-year period (1983–2014) to determine the occurrence, abundance, and diversity patterns of hydromedusae in the SWA. Analyses were made at both community and species levels. Two abundance hot spots of hydromedusae were identified, where values up to 2,480 ind. m^-3^ were recorded between 2003 and 2014. *Liriope tetraphylla* and *Obelia* spp. were the main responsible for recurrent peaks. Diversity indexes were in the range of those published for temperate areas worldwide, and some coastal zones showed values that can be considered moderate to high for a temperate neritic region. The community analysis yielded 10 groups following previously determined biogeographic schemes throughout the study area. This work enhances the knowledge of hydromedusae in the SWA and provides essential information about the current global warming context and the gelatinous zooplankton data necessity.

## Introduction

Gelatinous zooplankton (i.e., mostly medusae and ctenophores, commonly grouped as “jellyfish”) are critical components of marine ecosystems because they can shape food webs [1,2]. They are ubiquitous and voracious on a wide range of prey including fish [2], and they typically display events of massive occurrences over a variety of spatial and temporal scales, which might be attributable to rapid population increases or to physical forcing that aggregates individuals [3–5]. In addition to the ecological implications, aggregations of jellies can also have socioeconomic costs, such as the negative impact on fisheries, the water intake clogs in power and desalination plants, or the problems caused for public health and tourism [6].

Despite the stated importance of jellyfish, the worldwide knowledge about them is patchy. Furthermore, reliable baseline data and long-term time series to understand their population dynamics are scarce [7,8]. To elicit information concerning a community structure, distribution and abundance patterns are essential to evaluate the evolution of a given community in order to detect changes over time and space together with their potential causes and the underlying processes [9–11]. Fundamental research in jellyfish ecology is required to define baselines that will allow researchers to make retrospective analyses and to identify trends at different spatial and temporal scales. Nevertheless, to achieve this goal, some geographic areas need to be addressed. One of the ocean regions most poorly studied in terms of jellyfish ecological data is the Southwestern Atlantic (SWA) [12,13]. There, the knowledge of species composition and distribution is characterized by a noticeable space-time discontinuity [14,15], and studies focused on jellyfish abundance in the area are practically nonexistent in spite of some efforts that have been made (see [16] for a review).

Jellyfish variability over time and space is modulated not only by the life history of the organisms but also by the geographic setting, the physical environment, and the reciprocal action among them [3,4]. In medusa species with the typical metagenetic life cycle (i.e., alternation of benthic polyp stage and free sexual planktonic medusa), like most hydromedusae (Hydrozoa), the abundance of the medusa stage will be determined by the success of each of the preceding stages involved in the life cycle defined, in turn, by the interaction with the environment, including both biotic and abiotic factors. Temperature, salinity, food supply, light, and predation are closely associated to the reproduction and growth of polyps, the liberation of the medusa, and the release of the larva, thus highly influencing the magnitude of the medusa peak by acting on biological traits [2,3,17]. However, not all hydromedusan species show metagenetic life cycles with alternation of asexual and sexual phases (see [18]). The species of the orders Trachymedusae and Narcomedusae, such as *Liriope tetraphylla* and *Pegantha laevis*, respectively, among others, have holoplanktonic life cycles (i.e., lack of benthic stages), thus reducing the environmental modulation to only one phase (the pelagic medusa).

On the other hand, changes in physical properties of the environment may form and maintain jellyfish aggregations due to a combination of the effects of physical forces (e.g., circulation, fronts) and behavioral responses (e.g., phototropism), particularly at local scales, without necessarily reflecting locally increased reproduction [4]. Analyses of several long-term (8 to 100 years) trends in medusa populations demonstrate that their abundances vary with climate, often at decadal scales (reviewed in [19]), and also with human-driven changes (e.g., [6]).

Accumulations of gelatinous organisms around physical discontinuities (e.g., fronts) are commonly reported, particularly at local scales [4,20]. On the other hand, as previously stated, food availability is considered a crucial factor in the regulation of jellyfish occurrence and reproduction [3]; hence, zones of high abundance of hydromedusae are likely to be found within productive and retentive areas such as estuarine and tidal fronts. Considering both, a 31-year data set and a macroscale area (>1.9 million nm^2^; ~6.7 million km^2^), the temperate SWA (27°-56° S), the aims of the study were *i*) to analyze the occurrence, abundance and diversity patterns of hydromedusae (at both community and species levels) identifying areas of high abundances (abundance hot spots of hydromedusae) and *ii*) to investigate the existence of hydromedusan assemblages in the study area. Results were discussed considering the physical and biological context of the study area [21–26] and the life history traits of the most relevant hydromedusan species. Finally, the knowledge gaps and guidelines for future research on hydromedusae in the region were provided.

## Materials and methods

### Study area

**Fig 1:**
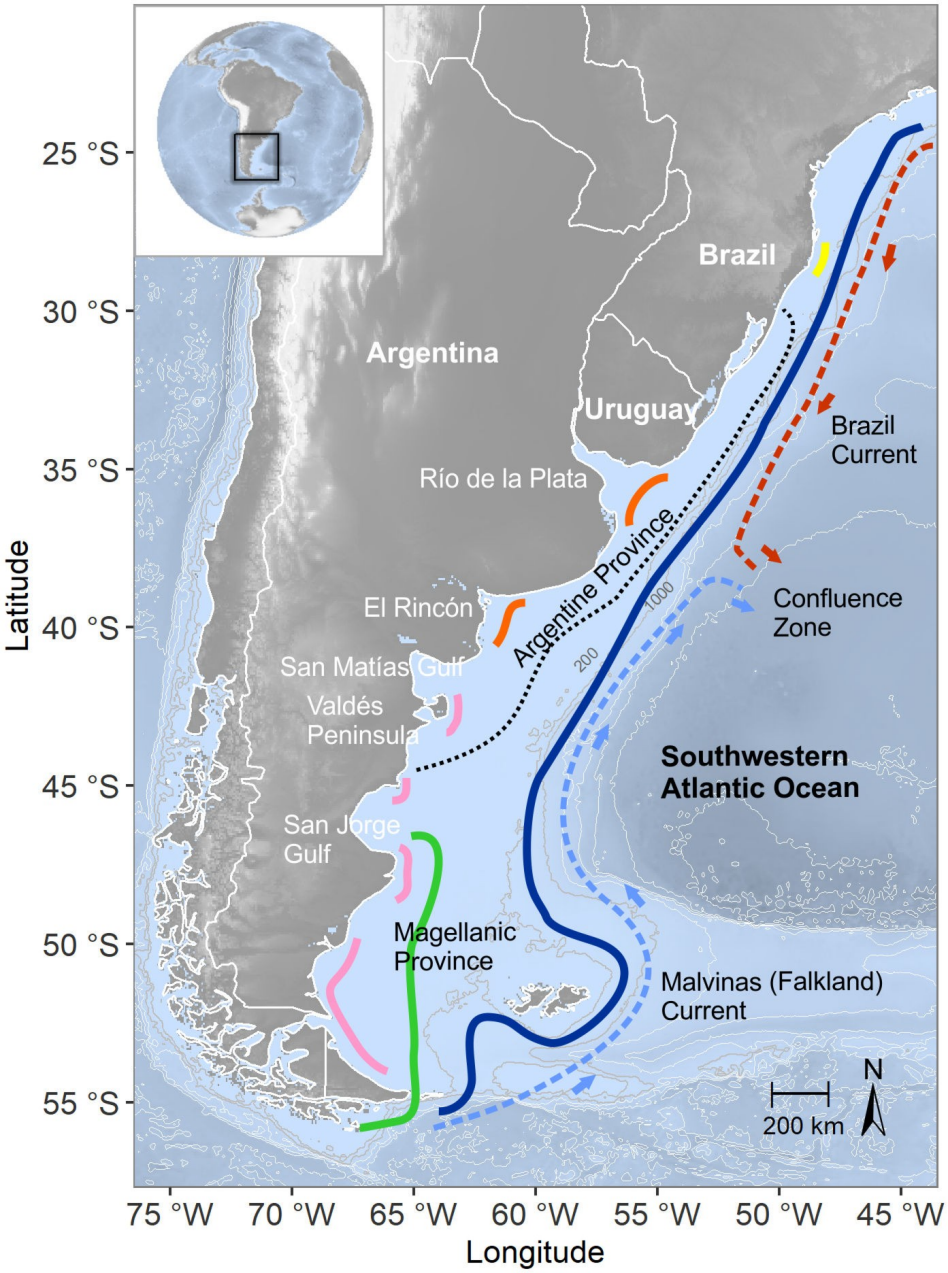
Study area. The thin dotted-black line represents the limit of the Argentine and Magellanic Provinces based on Balech and Erlich [27]. Dotted red and light blue lines represent the currents, and colorful lines correspond to the fronts in the region (yellow: South Brazil upwelling, orange: temperate estuarine fronts; pink: tidal fronts, green: Patagonian current front, blue: shelf-break front) in accordance with Acha et al. [21,22].

The study area comprised the continental shelves of southern Brazil, Argentina and Uruguay (27°-56°S; Fig 1). It is a complex region from a hydrographic viewpoint. It encompasses two biogeographic provinces (i.e., the Argentine and the Magellanic Provinces), which environmental features explain the differences in their faunistic composition, and diverse oceanographic structures that include several estuaries, water masses, wind systems, tidal regimens, and two major oceanic currents [21,25,27] (see Fig 1).

Regarding the biogeographic regions, the Argentine Province is associated with warm and temperate-cold coastal waters of intermediate salinities, movable sandy bottoms, and a marked heterogeneity of its faunistic components as a consequence of the mixed subtropical and sub-Antarctic features [27]. The Magellanic Province is characterized by sub-Antarctic waters and strong western winds, gravel bottoms, where large algae grow, and faunistic homogeneity and own taxa [27]. The shelf circulation consists of a southward flow of warm waters in the north, the Brazil Current, and a northward flow of cold waters in the south, the Malvinas (Falkland) Current [23]. The former consists of warm, salty, and oligotrophic waters, whereas cold, relatively low-salinity and nutrient-rich waters characterized the Malvinas (Falkland) Current, which occupies the wide sub-Antarctic shelf, south of *ca*. 38°S [24,25]. The collision of these two currents, near the mouth of the Río de la Plata (~ 38°S), creates a strong thermohaline front known as the Brazil/Malvinas Confluence, where intense mixing of sub-Antarctic and subtropical waters occurs along it [24,25]. The immediate portion north of 38°S is highly influenced by the freshwater discharge of the Río de la Plata and by its proximity to the Malvinas (Falkland)/Brazil Confluence Zone, which promotes energetic exchanges between shelf and deep ocean waters [25].

Intense frontal transitions in several coastal locations and along the shelf break promote vertical circulations and inject nutrients into the upper layer [21,25]. The study region is under a temperate climate regime, characterized by marked seasonality and the formation of strong thermoclines during spring and summer [21].

### Sample collection

To study the occurrence, abundance, and diversity patterns of hydromedusae in the SWA, a total of 3,727 zooplankton samples from this area was analyzed encompassing a 31-year period (from 1983 to 2014), an area of ~6.7 million km^2^, and a filtered volume of at least 244,157 m^3^. Zooplankton samples were collected during fishery research cruises carried out by the Instituto Nacional de Investigación y Desarrollo Pesquero (INIDEP, Argentina) covering most of the study area, and during plankton surveys performed by the Instituto Argentino de Oceanografía (IADO-CONICET/UNS, Bahía Blanca, Argentina) at a smaller spatial scale over the Bahía Blanca Estuary and the adjacent inner shelf called El Rincón.

**Fig 2:**
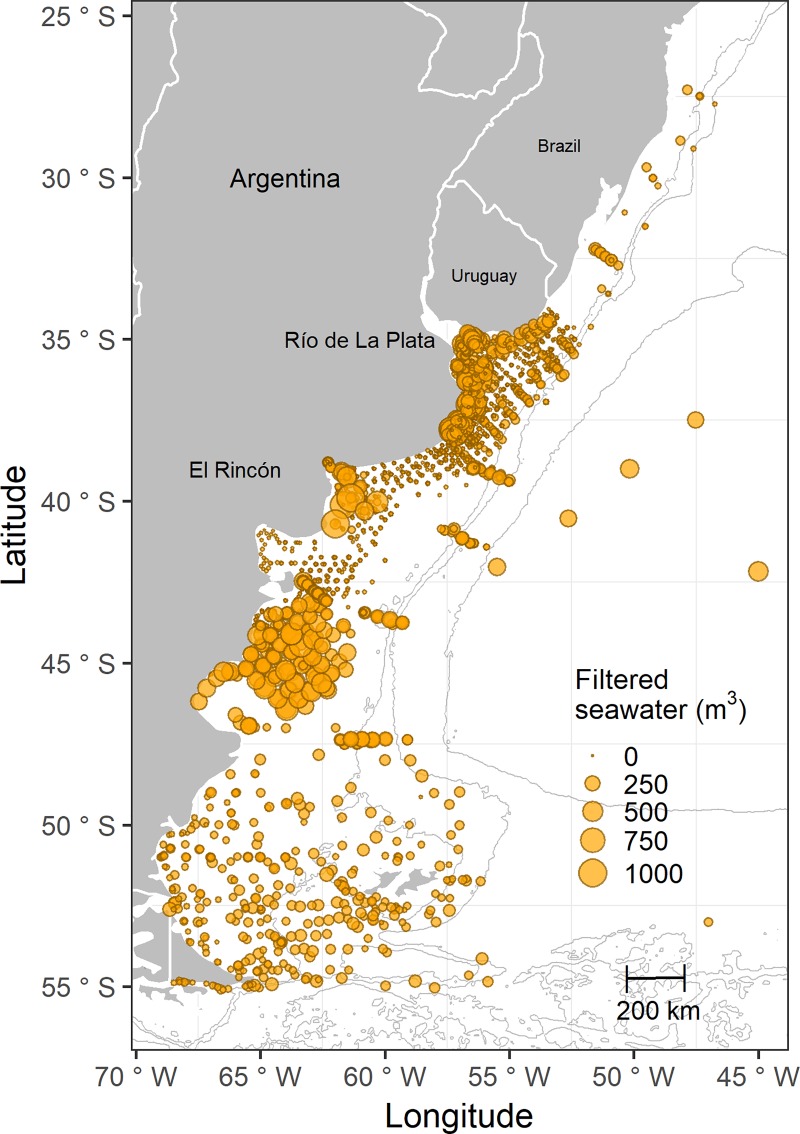
Spatial distribution of the filtered seawater (m^3^) along the temperate SWA (27°-56°S) during the period 1983–2014. Data is shown in a continuous scale (n = 3,770; min. value = 0.29 m^3^, max. value = 1,056.80 m^3^).

Samples were collected during daytime from late spring to summer (from October to March), and the effort of sampling was quite homogeneous in most of the study area (Fig 2). Samples were taken using a variety of plankton nets (Bongo, Nackthai, Motoda, Pairovet, Calvet, and Multinet) equipped with mesh sizes ranging from 200 to 500 μm (see [28] for descriptions of nets) and operated in oblique trawls from the proximity of the bottom to surface. Filtered volumes were estimated using flowmeters. Hydromedusae were separated from the samples, preserved in a 4% formalin-seawater solution, and stored at J. J. Nágera Biological Station (UNMdP, Mar del Plata, Argentina) and at IADO-CONICET/UNS (Bahía Blanca, Argentina).

### Sample examination

Hydromedusan specimens were identified and quantified under a stereomicroscopy. Abundance was expressed as ind. m^-3^. Species-level identification was mainly based on Bouillon [29] and Bouillon et al. [18,30], and synonymies on Oliveira et al. [31].

### Data analyses

#### Occurrence, abundance, and diversity patterns of hydromedusae

The full set of samples (3,727) was examined to determine the frequency of occurrence of hydromedusae as a percentage of the total samples. To analyze the spatial distribution of the occurrence, abundance, and diversity of hydromedusae, the data were cleaned (e.g., observations containing no hydromedusan data were eliminated), and a matrix of 1,063 observations containing hydromedusan data was examined. From this matrix, a frequency histogram was made to obtain the frequency of occurrence (%) of the hydromedusan species. The abundance of hydromedusae (total abundance and the abundance of key species) was plotted along the study area (spatial variation) and the study period (temporal variation) to explore the data distribution. To accomplish this, the study area was divided into one-degree grid squares to manage the data series and facilitate the visualization of patterns resulting in a 120-square matrix of 45 variables (i.e., species). This matrix was used for the calculation of the diversity indexes and for the community analysis (see Multivariate analyses) calculating the mean abundance of hydromedusae for each of square.

The richness (number of taxa, S), the Shannon-Wiener index:

$$H^{\prime }=-\sum pi\;\ln\;pi$$ (1)

where *pi* is the proportion of each taxon in the sample), and the Pielou´s evenness index:

J′=H′/lnS (2)

where S is the total number of taxa) were calculated in each square. In the mentioned index, H' increases as both the richness and the evenness of the community increase, and J' varies between 0 and 1, being maximum when all taxa present similar abundance value (absence of a dominant species). The indexes were plotted along the study area to visualize their distribution. Diversity index calculation was performed in R version 3.4.4 [32] using the Vegan packages [33]. Graphing was performed using the ggplot2 package [34] and the functionalities of the marmap package [35].

#### Hydromedusan community: Multivariate analyses

To test for the presence of sample groups in the set of samples (null hypotheses of “absence of structure”), the similarity profile routine (SIMPROF) was applied [36], followed by a hierarchical agglomerative clustering (CLUSTER) coupled with group-average linkage. This technique was based on triangular matrices using the Bray-Curtis similarity index on square root-transformed abundance data to enhance the contribution of the less abundant taxa [37]. Similarity percentage analysis (SIMPER) was then used to identify the taxa that were most responsible for the observed pattern. It examines the contribution of each taxon to the similarity within each group already detected by SIMPROF and to the dissimilarity between the groups. The more abundant *a* species is within *a* group, the more it contributes to the intra-group similarity; a species typifies a group if it is found at a consistent abundance throughout, so the standard deviation (SD) of its contribution is low [37]. Considering that taxa/species with high dissimilarity to standard deviation ratio (Diss/SD) are good discriminating ones (see [37]), those with (Diss/SD) > 2 were identified as discriminating hydromedusan taxa/species. The PRIMER 6 package was used to perform these analyses.

## Results

### Hydromedusan composition

In total, 45 hydromedusan taxa belonging to 24 families and 35 genera were identified. The five hydromedusan orders were represented by the following contributions: 42.2% Leptothecata, 35.5% Anthoathecata, 8.9% Trachymedusae, 6.7% Limnomedusae and 6.7% Narcomedusae. The list of the identified taxa/species is shown in Table 1.

**Table 1:** Hydromedusan taxa analyzed in the SWA (27°-56°S) from 1983 to 2014 in descending order based on their maximum abundance detected. Taxonomic details, mean abundance (Mean Ab., ind. m^-3^ ± SD), maximal abundance value (Max. Ab., ind. m^-3^), the year when the maximal abundance was detected, and the location of the maximum are given. N = 1,063 in all cases.

Order	Family	Genera or Species	Mean Ab. (ind. m^-3^)	Max. Ab. (ind. m^-3^)	Year	Location of the Max. Ab.
Trachymedusae	Geryoniidae	*Liriope tetraphylla*	16.81	2,474.49	2005	35°04'18.6"S 56°24'12.6"W
Leptothecata	Campanulariidae	*Obelia* spp.	3.69	1,578.82	2003	39°41'00.0"S 61°52'00.0"W
Leptothecata	Lovenellidae	*Eucheilota ventricularis*	0.7488	175.56	2004	35°19'00.0"S 55°34'00.0"W
Narcomedusae	Cuninidae	*Cunina octonaria*	0.0633	28.05	2006	37°20'00.0"S 56°50'00.1"W
Leptothecata	Campanulariidae	*Clytia hemisphaerica*	0.0689	21.60	2014	38°57'35.5"S 62°10'15.5"W
Anthoathecata	Corynidae	*Coryne eximia*	0.0473	21.50	2006	41°57'29.5"S 62°30'33.0"W
Anthoathecata	Corymorphidae	*Euphysa aurata*	0.0735	13.85	1994	41°50'00.1"S 61°00'00.0"W
Leptothecata	Blackfordiidae	*Blackfordia virginica*	0.0109	11.64	2000	36°18'00.0"S 56°44'00.1"W
Anthoathecata	Proboscidactylidae	*Proboscidactyla mutabilis*	0.1871	10.00	2004	38°24'00.0"S 57°07'00.0"W
Leptothecata	Mitrocomidae	*Mitrocomella brownei*	0.0703	9.46	2006	39°09'00.0"S 60°42'42.1"W
Leptothecata	Laodiceidae	*Laodicea undulata*	0.0257	5.09	2004	42°08'00.0"S 63°20'00.0"W
Leptothecata	Phialellidae	*Phialella falklandica*	0.0046	4.90	1996	50°17'00.2"S 68°34'00.4"W
Anthoathecata	Corymorphidae	*Corymorpha januarii*	0.0032	2.12	2006	39°20'24.0"S 61°27'00.0"W
Anthoathecata	Pandeidae	*Amphinema dinema*	0.0034	2.08	2000	38°13'12.0"S 57°27'00.0"W
Trachymedusae	Rhopalonematidae	*Rhopalonema velatum*	0.0146	2.06	2006	53°37'04.8"S 64°08'35.4"W
Anthoathecata	Bougainvilliidae	*Bougainvillia pagesi*	0.0015	1.54	2006	37°30'34.8"S 57°08'00.0"W
Leptothecata	Mitrocomidae	*Cosmetirella davisi*	0.0066	1.35	2003	36°44'33.7"S 55°28'00.0"W
Leptothecata	Eirenidae	*Eutonina scintillans*	0.0017	1.13	2006	38°53'54.1"S 60°04'14.4"W
Anthoathecata	Bougainvilliidae	*Bougainvillia macloviana*	0.0025	1.09	1994	49°59'05.4"S 67°36'24.0"W
Anthoathecata	Tubulariidae	*Hybocodon chilensis*	0.0030	1.02	2006	43°40'11.5"S 64°09'36.0"W
Leptothecata	Aequoreidae	*Aequorea forskalea*	0.0009	0.90	2014	39°00'08.1"S 61°16'59.8"W
Leptothecata	Campanulariidae	*Clytia gracilis*	0.0044	0.87	2004	42°21'00.0"S 63°02'00.0"W
Anthoathecata	Oceaniidae	*Turritopsis nutricula*	0.0026	0.68	2004	41°17'00.0"S 62°50'00.1"W
Leptothecata	Campanulariidae	*Clytia lomae*	0.0015	0.66	2004	37°57'00.0"S 56°45'00.0"W
Anthoathecata	Pandeidae	*Leuckartiara octona*	0.0022	0.64	2006	37°06'09.0"S 56°37'52.9"W
Limnomedusae	Olindiidae	*Gossea brachymera*	0.0024	0.58	2013	38°57'35.5"S 62°10'15.5"W
Anthoathecata	Hydractiniidae	*Podocoryna borealis*	0.0008	0.56	2006	41°19'58.8"S 64°49'54.1"W
Trachymedusae	Rhopalonematidae	*Aglaura hemistoma*	0.0014	0.39	2004	31°05'07.2"S 50°22'07.2"W
Narcomedusae	Solmundaeginidae	*Solmundella bitentaculata*	0.0005	0.32	1999	37°00'24.0"S 55°17'42.0"W
Anthoathecata	Tubulariidae	*Hybocodon unicus*	0.0005	0.27	1999	54°29'08.8"S 61°28'28.5"W
Leptothecata	Campanulariidae	*Clytia simplex*	0.0010	0.24	2006	37°54'00.7"S 56°39'08.5"W
Anthoathecata	Hydractiniidae	*Podocoryna tenuis*	0.0003	0.16	1996	54°52'00.6"S 68°16'00.2"W
Anthoathecata	Corymorphidae	*Corymorpha gracilis*	0.0001	0.11	2004	31°05'07.2"S 50°22'07.2"W
Leptothecata	Mitrocomidae	*Mitrocomella frigida*	0.0002	0.11	1996	53°25'00.3"S 65°00'00.0"W
Leptothecata	Laodiceidae	*Laodicea pulchra*	0.0002	0.09	2010	44°25'27.9"S 64°58'45.1"W
Leptothecata	Lovenellidae	*Mitrocomium cirratum*	0.0001	0.08	2004	27°43'31.2"S 46°45'05.4"W
Leptothecata	Tiarannidae	*Krampella* sp.	0.0001	0.07	1996	54°55'00.7"S 67°10'00.1"W
Limnomedusae	Olindiidae	*Olindias sambaquiensis*	0.0000	0.05	1995	36°17'06.0"S 56°33'16.1"W
Anthoathecata	Rathkeidae	*Rathkea formosissima*	0.0000	0.04	1996	54°52'00.6"S 68°16'00.2"W
Anthoathecata	Pandeidae	*Amphinema rugosum*	0.0001	0.04	1996	55°06'00.1"S 66°30'00.4"W
Leptothecata	Mitrocomidae	*Halopsis ocellata*	0.0001	0.04	1996	54°10'00.1"S 64°55'00.7"W
Limnomedusae	Olindiidae	*Aglauropsis kawari*	0.0001	0.02	2006	36°26'39.0"S 56°40'55.8"W
Narcomedusae	Solmarisidae	*Pegantha laevis*	0.0001	0.02	2001	54°46'28.8"S 63°12'30.2"W
Trachymedusae	Rhopalonematidae	*Sminthea eurygaster*	0.0000	0.01	1999	53°54'21.1"S 62°55'12.7"W
Leptothecata	Tiarannidae	*Modeeria rotunda*	0.0000	0.01	1999	54°29'07.0"S 62°11'18.1"W

### Occurrence, abundance and diversity patterns of hydromedusae

**Fig 3:**
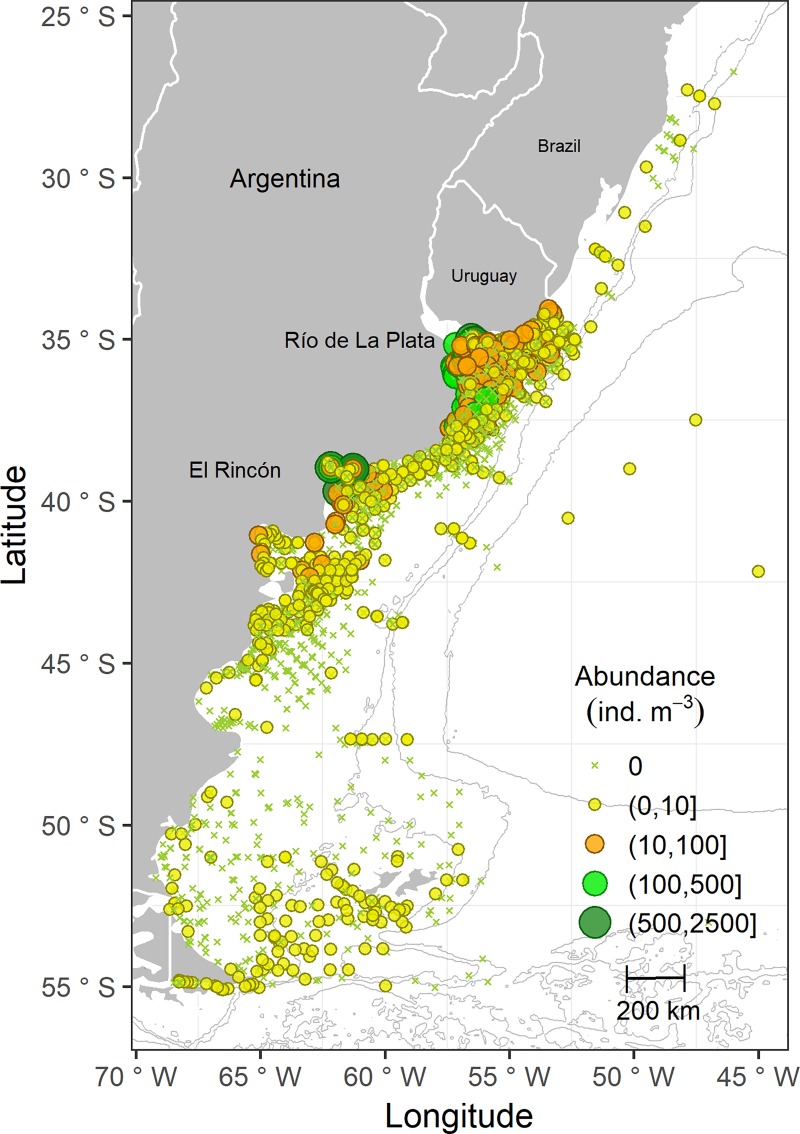
Spatial distribution of the abundance of hydromedusae (ind. m^-3^) in the SWA (27°-56°S) from 1983 to 2014. Data is shown in ranges (n = 3,727).

Regarding the frequency of occurrence, 33.6% of the full set of samples along the 31 years contained hydromedusae. The most frequent taxa were *L*. *tetraphylla* (37.5%), *Proboscidactyla mutabilis* (26.7%), *Obelia* spp. (20.8%), *Eucheilota ventricularis* (14.7%), *Rhopalonema velatum* (8.4%), and *Clytia hemisphaerica* (5.3%). The remaining taxa (n = 39; see Table 1) occurred in frequencies lower than 5%. Fig 3 shows the distribution of the abundance of hydromedusae. The highest abundances (> 100 ind. m^-3^) were represented by 4.4% of the samples. North of 33° S (northern Uruguayan coast—southern border of Brazil), and south of 41° S (northern Patagonia, Argentina), abundances did not exceed 24 ind. m^-3^. Between these two areas, we identified two hot spots, defined as the areas with the highest abundances of hydromedusae, which corresponded to the Río de la Plata Estuary (33°-37°S, 53°-59°W) and El Rincón region (38°-41°S, 61°-63°W) (Fig 3). Hydromedusa abundances of more than 100 ind. m^-3^ were detected 33 and 10 times in each of these areas, respectively, along the study period, including maximum values of more than 1,000 hydromedusae per m^3^. These maximum abundances were practically monospecific, caused by *L*. *tetraphylla* (2,480 ind. m^-3^ in the Río de la Plata Estuary and 1,778 ind. m^-3^ in El Rincón region) and *Obelia* spp. (1,579 ind. m^-3^ and 1,199 ind. m^-3^, both in El Rincón region) (Fig 3), and detected between 2003 and 2014. A high proportion of these nearly monospecific maximums were juveniles (i.e., immature medusae) of the mentioned species.

**Fig 4:**
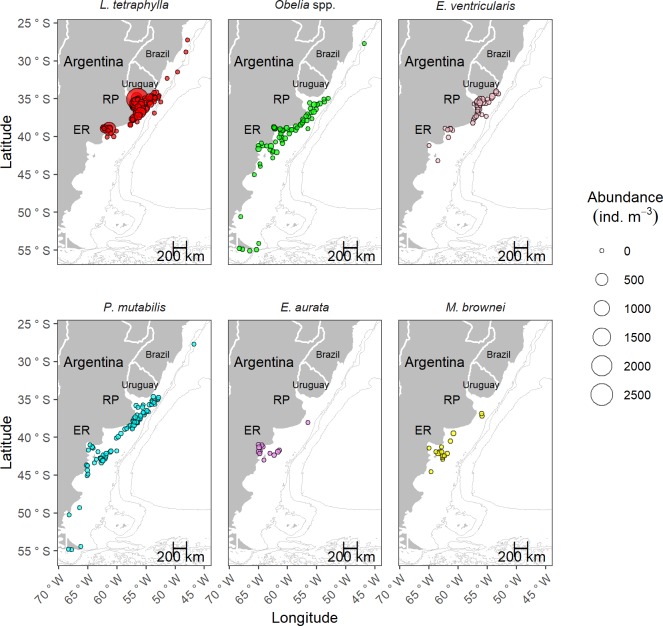
Spatial distribution of the abundance (ind. m^-3^) of the six most abundant hydromedusan taxa/species analyzed (in descending order of abundance: *Liriope tetraphylla*, *Obelia* spp., *Eucheilota ventricularis*, *roboscydactila mutabilis*, *Euphysa aurata*, and *Mitrocomella brownei*) along the SWA (27°-56°S) from 1983 to 2014. Data is shown in a continuous scale (min. value = 0.0017 ind. m^-3^, max. value = 2,474.49 ind. m^-3^). RP: Río de la Plata, ER: El Rincón.

Concerning species levels, the six most abundant taxa were *L*. *tetraphylla*, *Obelia* spp., *E*. *ventricularis*, *P*. *mutabilis*, *Euphysa aurata*, and *Mitrocomella brownei* (Fig 4 and Table 1). The remaining taxa displayed maximums below 9 ind. m^-3^, being most of them found in one of the two identified hot spots (Table 1).

**Fig 5:**
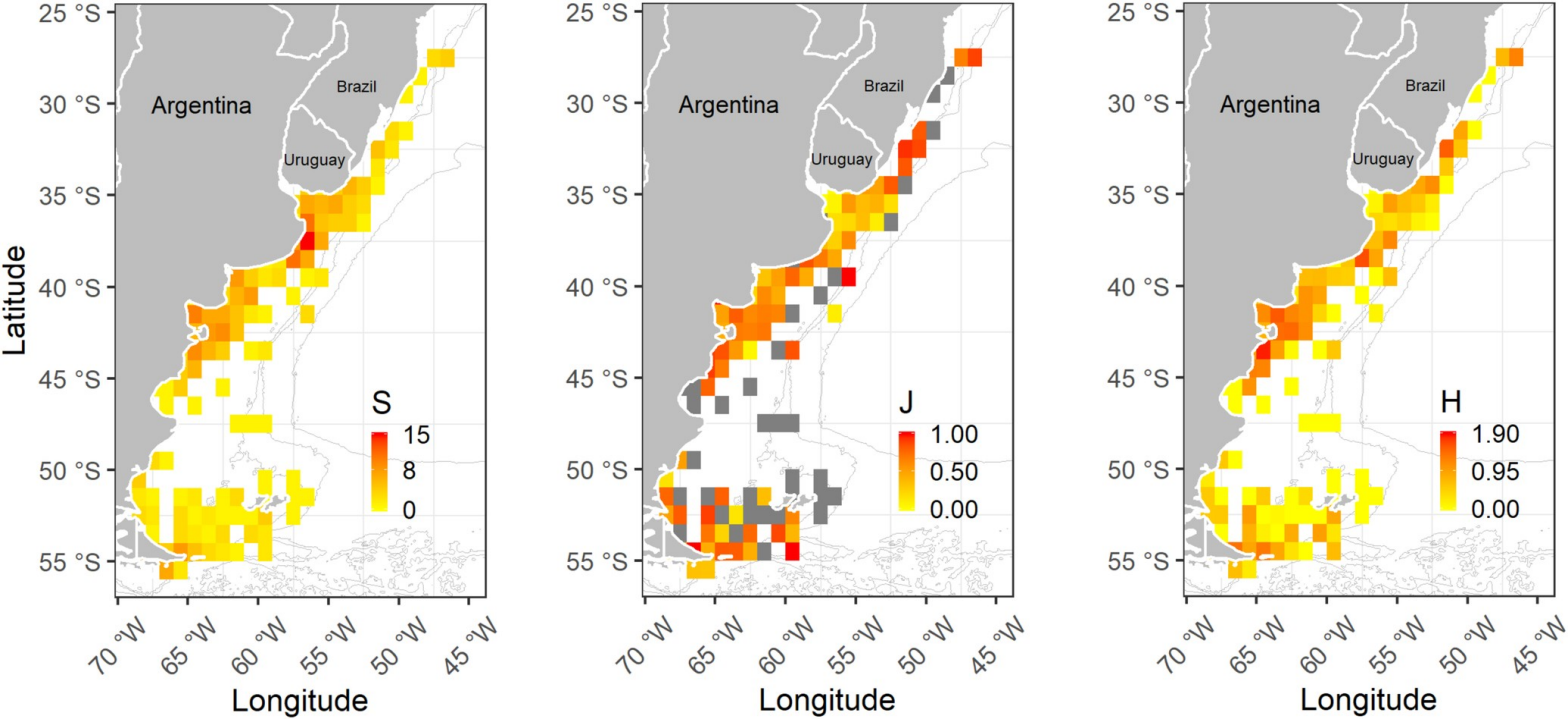
Spatial distribution of richness (S), equitability (J'), and diversity (H') of hydromedusae along the SWA (27°-56°S).

The highest richness values were found in coastal waters of the central zone of the study area (S = 15 at 37°30' S 56°30' W; S = 11 at 38°30' S 57°30' W and 36°30' S 56°30'). Maximums of equitability (J' = 1) were found over the 200 m isobath, overlapping the shelf-break front (Figs 1 and 5). The highest diversities were detected in coastal waters of northern Patagonia (H' = 1.85 at 43°30' S 64°30' W; H' = 1.6 at 41°30' S 63°30' W), in coastal waters of the central zone of the study area (H' = 1.6 at 38°30' S 57°30' W), and in the south of Brazil (H' = 1.5 at 32°30' S 51°30' W), in the northern border of the study area (Fig 5).

### Hydromedusan assemblages

**Fig 6:**
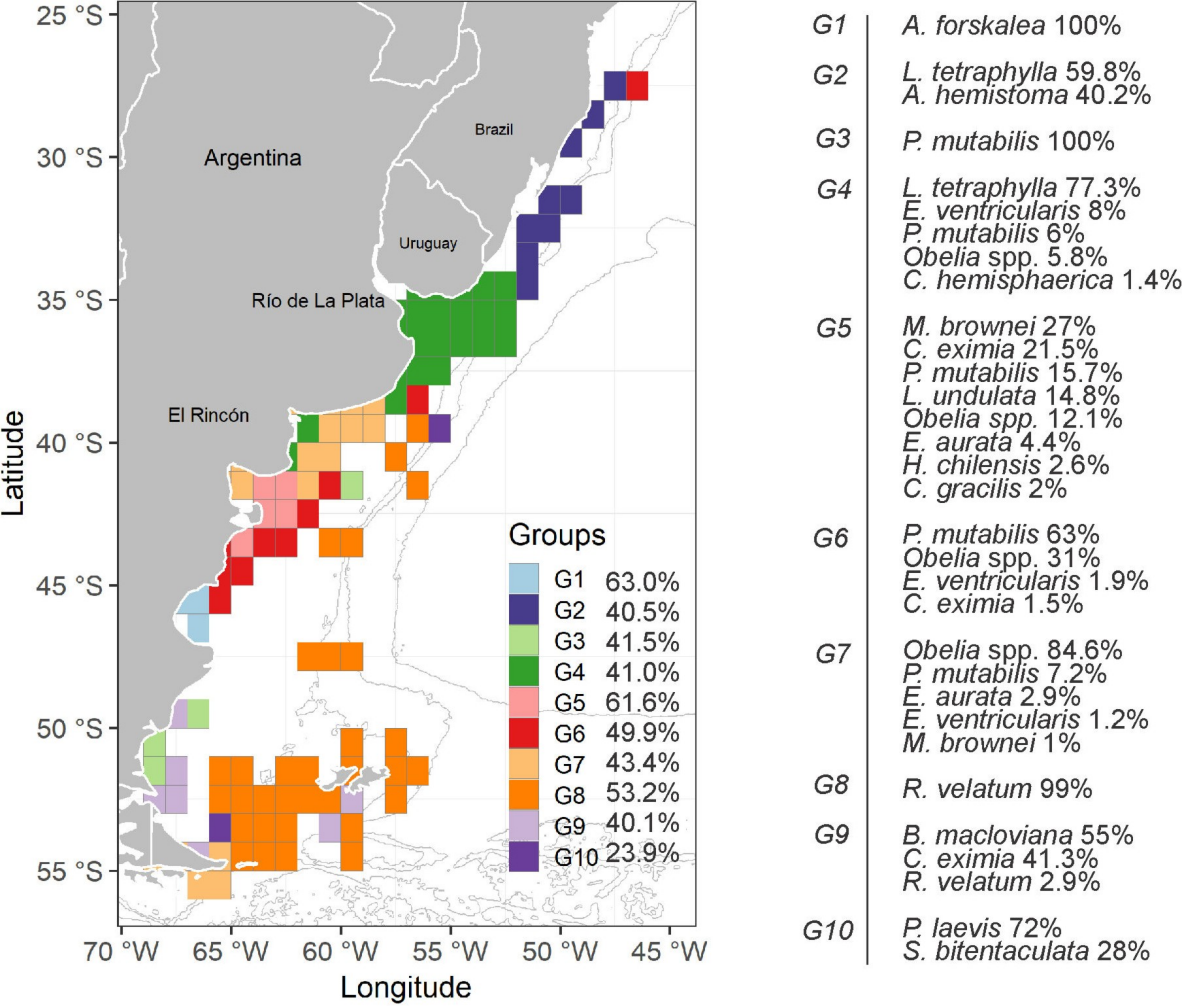
Community analysis of hydromedusae in the temperate SWA (27°-56°S) by multivariate techniques resulting from a dendrogram for hierarchical clustering of 120 1°x1° squares, using group-average linking of Bray-Curtis similarities calculated on root square-transformed abundance data. Cluster was superimposed on the map of the study area showing the groups detected by SIMPROF (G 1—G 10). Similarity Percentages (SIMPER) showing the average similarity of each group, and the hydromedusan taxa which contributed most (%) to the similarity within each group is also shown.

Ten groups of hydromedusae (G 1—G 10) were detected in the data matrix by squares (SIMPROF: Pi = 2.74, p = 0.01) and displayed by the dendrogram for hierarchical clustering based on similarities (Fig 6). From the analyzed squares, 95.8% were grouped, and five squares were left ungrouped (uncolored squares in Fig 6). The G 1 group (average similarity: 63%) encompassed three squares on the San Jorge Gulf (45° S; 65° - 66° W), where *Aequorea forskalea* accounted for 100% of the similarity. The G 2 group (average similarity: 40.5%) consisted of nine squares in a transitional zone between temperate and subtropical waters where the neritic zone narrows, in the southern coast of Brazil; two species, *L*. *tetraphylla* and *Aglaura hemistoma* accounted for 59.8% and 40.2% of the similarity there, respectively. The G 3 group (average similarity: 41.5%) consisted of four squares mainly on coastal southern Patagonian waters formed by the contribution of *P*. *mutabili*s. The G 4 group (average similarity: 41%) was consisted of 23 squares covering the estuarine zone of the Río de la Plata and El Rincón; although 17 taxa were detected, only five taxa contributed most to its formation, being *L*. *tetraphylla* the species that accounted for the highest contribution (77.3%) (Fig 6). The G 5 group (average similarity: 61.6%) consisted of five squares and covered the continental shelf outside the San Matías Gulf and the Valdés Peninsula zone (41° - 43° S) and, although 45 taxa were detected, only eight taxa contributed most to its formation, being *M*. *brownei* and *Coryne eximia* the main contributing species (Fig 6). The G 6 group (average similarity: 49.9%) included 10 squares mainly along the front of the slope and the southern area of the Patagonian continental shelf (between 38° and 46° S); nine taxa were detected, but only four hydromedusan taxa contributed most to the formation of this group; *P*. *mutabilis* contributed with 63% (Fig 6). The G 7 group (average similarity: 43.4%) consisted of 18 squares and covered the coast in the central study area (including the outside region of El Rincón and the San Matías Gulf) and the southern coastal waters; although 18 taxa were detected, five contributed most to its formation, being *Obelia* spp. the most important contributor (84.6%; Fig 6). The G 8 group (average similarity: 53.2%) consisted of 32 squares along the front of the slope and the south of the Patagonian continental shelf (between 40° and 55° S); eight species were detected, but *R*. *velatum* contributed almost entirely to its formation (99.9%). The G 9 group (average similarity: 40.1%) consisted of seven dispersed squares along the southern platform (49° - 55° S); four species were detected, but it was mainly formed by *Bougainvillia macloviana* and *C*. *eximia* (Fig 6). The G 10 group (average similarity: 23.9%) consisted of two dispersed squares; *P*. *laevis* and *Solmundella bitentaculata* (72 and 28%, respectively) contributed to their formation (Fig 6).

Dissimilarity among the groups was high, differing in 34 out of the 45 group pairs (76%) by more than 90% of dissimilarity. The discriminating hydromedusan taxa according to values of Diss/SD ratio were *M*. *brownei*, *L*. *tetraphylla* and *Obelia* spp., *P*. *mutabilis*, *Laodicea undulata* and *C*. *eximia*, and *R*. *velatum*.

## Discussion

### Hydromedusan patterns over the temperate SWA

At a macroscale analysis, the study area (27° - 56°S) encompasses two hot spots in terms of frequency of occurrence and abundance of hydromedusae. Considering both the long temporal scale analyzed, which allows us to perceive patterns (i.e., arrangements repeated in time and space) and the results of local samples (e.g., detection of immature planktonic stages), these medusa hot spots may be associated not only to retentive hydrographic features (apparent bloom), but also to population-level changes (true bloom) (see [4,5]), at least, in some occasions.

The two abundance hot spots of hydromedusae identified in our study (the Río de la Plata Estuary and El Rincón region) include two large estuarine fronts of particular hydrographic and topographic features within a zone defined by high biological production [21,22]. Considering the abundance patterns and the assemblages reported by the multivariate analyses, the covered temperate estuaries seem to be particularly proper for the development of *L*. *tetraphylla*, although other hydromedusan species were also detected and are discussed below. *Liriope tetraphylla* is widely distributed in warm waters of all oceans [38] and frequently observed in coastal ecosystems worldwide displaying, at times, higher abundances than in the open sea [39–43]. In southern Brazil and the Río de la Plata estuarine area, *L*. *tetraphylla* peaks seasonally [20,44,45]; however, the abundance peak recorded in our study (2,475 ind. m^-3^) outnumbers the abundance values reported for *Liriope* from SWA waters, surpassed only by the one indicated by Mianzan et al. [46] at coastal waters to the south (~38°S) under an aggregation and true bloom condition. The proliferation of *L*. *tetraphylla* at both estuaries may be boosted by the high productivity of the areas [21,22,26] and also promoted by the traits and plasticity of the species. It adapts to a wide range of environmental variables, such as salinity and temperature [39,42], and can take advantage of food availability due to its high predation rates on a diverse food spectrum (e.g., copepods, fish eggs and larvae, chaetognaths) [47–49]. These traits may enable the species to achieve rapid population increases (see [42]). The occurrence of *L*. *tetraphylla* in El Rincón region and massive accumulations in recent years reveal that its abundance distribution is expanding southwards [50]. Changes in the latitudinal distribution and abundance patterns of the species are one of the signals of climate warming, and warm-water species abundances would be positively favored [10,19,51,52]. Indeed, rising temperatures and drier conditions have been recorded in the Bahía Blanca Estuary affecting the plankton communities (i.e., producing changes in phenology and composition) via alterations of the water chemistry and predator-prey interactions [53,54]. This current environmental scenario may support the underlying warming process that could be modulating the observed distributional expansion of *L*. *tetraphylla*. Further studies are needed in order to confirm this hypothesis.

On the other hand, when addressing the ecological context, in addition to the role of gelatinous organisms as important plankton predators, their role as potential prey to fish should be considered [55]. Thirty-nine fish species in the SWA are reported to eat jellyfish [56]. In fact, in both detected abundance hot spots, we found high prevalence of endoparasites inside *L*. *tetraphylla* and other common hydromedusae, such as *E*. *ventricularis* and *C*. *hemisphaerica* (this study), which culminated their life cycles in fish (i.e., *Monascus* and *Opechona* genera). This finding disclosed the trophic role as fish prey these hydromedusae have in the regional pelagic webs because the mentioned endoparasites were observed in the fish species studied in the region (see [57,58]). In addition to the potential ecological implications of *L*. *tetraphylla*, this hydromedusa also involves sanitary concerns because, under high density conditions (locally known as “tapioca”), it causes dermatitis, paresthesia and burning irritation [46]. Taken together, the ecological and socioeconomic implications, and the responsiveness to climate warming support the relevance of the continuity of the studies on the population pattern dynamics of this species, adding experimental observation on ecological roles and environmental modulation.

Also relevant was *Obelia* genus, a meroplanktonic widely distributed genus commonly found in shallow coastal ecosystems [41,59–61], where it can display exceptional abundances (25,800 ind. m^-3^ close to freshwater influence, see [62]). In SWA waters, *Obelia* medusae had been previously reported in the San Matías Gulf reaching abundances of 43,530 ind. 1,000 m^-3^ [63]. In our study, they were frequent and displayed higher accumulations, particularly in the Bahía Blanca Estuary (> 1,000 ind. m^-3^; Figs 3 and 6). Because *Obelia* species cannot be reliably identified if they are not linked to their life cycles [18], we just referred to the genus in this study, although at least five *Obelia* species inhabit the temperate SWA [14,64–66]. The observed *Obelia* peaks in El Rincón region may be attributed to *Obelia longissima*, which had been previously recorded in the area displaying a true bloom following a massive hydroid shoreline accumulation event [64]. However, *Obelia dichotoma*, *Obelia longa*, and *Obelia bidentata* also occur in the mentioned coastal area [65], and these three species, along with *Obelia geniculata*, were also reported farther south, on the Patagonian coasts [66]. The coexistence of several *Obelia* species encompassing temperate and sub-Antarctic coastal waters may result in the observed assemblage led by this genus (G 7 group; Fig 6). The abundance pattern displayed by *Obelia* medusae may be driven, in part, by their apparent and unusual trophic preference. *Obelia* medusae have a peculiar architecture [59] that determines distinct swimming and trophic behaviors, enabling them to specialize on nano- and microplankton consumption [67,68]. These preys are highly available on coastal environments, and certain bacteria and flagellates can be particularly high on this kind of habitat, subjected to the synergic effect of climate and anthropogenic pressures as indicated by López Abbate et al. [69,70] for the Bahía Blanca Estuary in El Rincón region. *Obelia* hydroids, on the other hand, may be benefited by both natural (biotic and abiotic) and man-made substrates [64,68] and by the availability of organic matter and microplankton as food resource [71]. The turbid, shallow, and eutrophic coastal ecosystems encompassed by El Rincón region may widely cover these requirements [50,65,72,73]. Furthermore, this area seems to enclose a proper environment not only for *Obelia* but also for other meroplanktonic hydromedusan species that may have similar environmental needs [50,65,72,74].

### Hydromedusan assemblages in the SWA

The regional patterns observed in our study were in accordance to the biogeographic distribution of hydromedusan species in the region (see [14,75]). As stated by Rodriguez et al. [75], the distributional patterns of hydromedusae in the temperature SWA were related to neritic water masses coinciding with previously suggested biogeographical provinces (i.e., the South Brazilian, the Argentine, and the Magellanic Provinces) ([27], see Fig 1).

Analyzing from North to South, the assemblages found in the southern border of Brazil (G 2 group) and the estuarine areas of the Río de la Plata and El Rincón (G 4 group) are associated with tropical waters (warm and salty) and South Atlantic central waters (temperate waters with salinities lower than 36, [25]), respectively. The fact that *L*. *tetraphylla* was the main contributor to both groups, highlights its plasticity and points out its distributional range, which reaches up the ~40° S in the SWA. *Aglaura hemistoma*, mainly associated to warm waters in the SWA, particularly to the tropical water mass of the Brazil Current [43,45], was absent in the G 4 group and those found southward in colder waters, in accordance with Rodriguez et al. [75]. Continuing south, the finding of the assemblages composed by the contributions of many meroplanktonic hydromedusae (G 5, G 6, and G 7 groups) was in accordance with the environmental and faunistic features of the transitional neritic zone such as the Argentine Province [27]. Furthermore, the results shown by the G 7 group coincided with previous research in the San Matías Gulf [63]. The particular hydrodynamic and bathymetric conditions of the San Matías Gulf, which provide confinement, seem to propitiate the development of a particular hydromedusan community, which differ from that one inhabiting the adjacent continental shelf [63]. The placement of the G 1 group (fully composed by *A*. *forskalea*) in the San Jorge Gulf (within the Magellanic Province) agreed with previous observations on the distribution of this species, which is commonly related to cold coastal waters [14,76]. The location of the G 8 group, which broadly followed the slope and was entirely composed by *R*. *velatum*, matched with previous biogeographic studies [14, 75] and with the oceanic feature that this holoplanktonic hydromedusa is associated with [77,78]. The austral assemblages, G 9 and G 10 groups, located mainly in the Magellanic Province and on the slope, correspond to hydromedusan species associated to sub-Antarctic waters [78,79]. The geomorphological features of this area facilitate the growth of different species of macroalgae [27] allowing meroplanktonic austral hydromedusae to use them for their hydroid settlement [66, 80]. Finally, the segregation of the G 3 group (entirely composed by *P*. *mutabilis*) from the G 6 group (mainly composed by *P*. *mutabilis*), could be due to a methodological artifact because of the different contribution of this highly recorded hydromedusan species over the South American Atlantic and Chilean Seas [14,75,81] to each of these groups.

In addition to the regional patterns, some isolated, although relevant findings, should be addressed. Besides the endemic hydromedusan species found to the SWA (*P*. *mutabilis*, *Olindias sambaquiensis*, and *Mitrocomella frigida* [14]), an exotic species was observed confirming its presence in the Río de la Plata estuarine area. In 2000, more than 5,000 individuals per sample of the invasive *Blackfordia virginica* were found in this zone, being its first record for the Argentine Sea (~36.2°S, [82]). This species was found again in 2006 at ~36°S (see Table 1) close to the location of its first record and recently recorded in Uruguayan waters (Vidal, Pers. comm.), suggesting that its introduction in the Río de la Plata estuarine area was successful as it happened in northward tropical and subtropical estuaries (see [83]). *Aequorea forskalea* entails another point to discuss. As mentioned before, this is a common hydromedusa frequently found in northern Patagonia [14,76], but it has been observed from 2014 onward forming dense aggregations, which lasted several days, in northern coastal waters, at 38.9°S [50]. Further and time-sustained research is needed to approximate to a scientific-based explanation for this observation. On the other hand, *Aequorea* medusae are too large (> 15 cm bell diameter) to be captured efficiently by plankton net trawling, which was the mostly used instrument in this study. Therefore, abundances observed in the present study seemed to be strongly underestimated. This assumption is supported by the frequent observation of high abundances captured through fish demersal trawls in northern Patagonia (the San Jorge Gulf) [76,84].

Regarding diversity indexes, our values are in the range of those published for temperate areas worldwide [41,61,81,85]. However, some coastal areas, such as the one at ~38°S and the Valdés Peninsula zone, emerge as relevant areas in relation to richness and diversity of hydromedusae, whose values can be considered moderate to high for a neritic temperate region [81,85,86]. At a regional scale, it is difficult to make comparisons, because hydromedusan diversity information is limited to few isolated values for certain sites within the SWA. Previous work focused on jellyfish research in the San Matías Gulf and in the Bahía Blanca Estuary and El Rincón (see Fig 1) revealed higher hydromedusan richness values than ours, pointing out the potential that these zones may have for jellyfish investigation (S = 23; see [50,63]). Considering that our study area is mostly coastal, it is not surprising that the highest diversities were found in coastal environments and, consequently, were mainly associated with the contribution of meroplanktonic hydromedusan species (particularly those belonging to Anthoathecata and Leptothecata orders) [38]. This result is in accordance with hydromedusan patterns observed in coastal ecosystems at different latitudes worldwide [10,41,45,85–89]. The hydrological dynamics and geomorphology of coastal environments would favor the development of hydromedusan species with benthic phases because costs are usually associated to retentive processes, which may favor reproductive encounters, and provide a wide offer of substrata, which enhances the probability of polyp settlement and success (see [66,90]).

### Final remarks, knowledge gaps and guidelines for future work

Because our database comes from fisheries stock assessment surveys, which have historically provided a cooperative platform for zooplankton works in Latin-American waters, it contains several potential sources of errors and noise. Samples were taken over a 31-year period along a huge geographical area by different operators, different sampling gears, and different mesh sizes, without neglecting the inherent noise of zooplankton data associated with the patchy distribution of organisms (e.g., [91]). However, the resulting spatial pattern can be understood based on current knowledge on the oceanography and biology of the region. In this way, weaknesses become strengths: the signal is strong enough to overcome data constraints so that the analysis produced coherent patterns. To focus on jellyfish is a crucial point for an appropriate assessment of the group. Certain areas have been already endorsed as more interesting in hydromedusan fauna than previously thought when jellyfish research was the purpose of the study and not the complement of other scientific investigation (see [50,63,65,72]). In addition, new hydromedusan species may be identified when morphological and molecular approaches were applied (see [88]).

We currently know which, how many, and where hydromedusan species are in the neritic environments of the SWA; this work not only provides the baseline for further studies on the hydromedusan fauna in the region but also offers valuable fundamental information to a global need of jellyfish data in the world oceans. In this regard, our data suggests that the most frequent and abundant species, *L*. *tetraphylla* and *Obelia* spp., could be increasing their abundances in the two identified hot spots, and the former also extending its abundance distributional range in response to changes in the temperature regime. However, these trends need further analyses and monitoring to be confirmed. Monitoring sustained programs are needed to continue studying the community and the pattern of abundance of hydromedusae, particularly of those hydromedusae that were detected as blooming ones and/or are associated to socioeconomic impacts (e.g., *Obelia* spp., *Aequorea* spp., *L*. *tetraphylla*, *O*. *sambaquiensis*).

Taxonomic certainty and knowledge about life cycles are strongly needed to get more accurate species identification and understanding. As stated by Cepeda et al. [26], under the current global warming, it has become practically mandatory to determine the number of species and their distribution borders to evaluate further possible biogeographical changes. Without a proper description of biodiversity and its functioning over time, it is difficult to ensure appropriate ecosystem management. Our results have settled a baseline for further studies on the hydromedusan diversity, abundance, and spatial distribution related to, for instance, climate change. The importance of the roles that hydrozoan key species play in the ecosystem functioning also has to be addressed. To accomplish this, future directions should focus on population dynamics (growth/mortality rates) and life history traits such as life cycles, reproductive biology, diet and feeding strategies.

## Conclusions

This is the first study that analyzed patterns of hydromedusae on a macroscale area (~6.7 million km^2^) with more than 3,700 analyzed plankton samples. The long-term study of the spatial distribution of the abundance and diversity of hydromedusae over the temperate Southwestern Atlantic pointed out specific clustering and abundance hot spots that yielded relevant values and, therefore, deserve attention. In ecological terms, few hydromedusan taxa make the difference showing important abundances in few sites along this macroscale area. Both, trachymedusa *L*. *tetraphylla* and leptomedusa *Obelia* spp. displayed abundances which surpassed, for instance, 1,000 ind. m^-3^ in two estuarine and productive areas in the central part of the study region (Río de la Plata and El Rincón). Our work improves the knowledge of hydromedusae in one of the ocean regions most poorly studied in terms of jellyfish ecological data, and provides relevant data in the light of the current debate on the increasing abundance of jellyfish in the open and coastal ocean.
