# Correction: Macroscale abundance patterns of hydromedusae in the temperate Southwestern Atlantic (27°–56° S)

**DOI:** 10.1371/journal.pone.0229678

**Published:** 2020-02-20

**Authors:** María Sofía Dutto, Carlo Javier Chazarreta, Carolina Soledad Rodriguez, Agustín Schiariti, Luciana Mabel Diaz Briz, Gabriel Néstor Genzano

Fig 4 is incomplete. The authors have provided a corrected version here.

**Fig 4:**
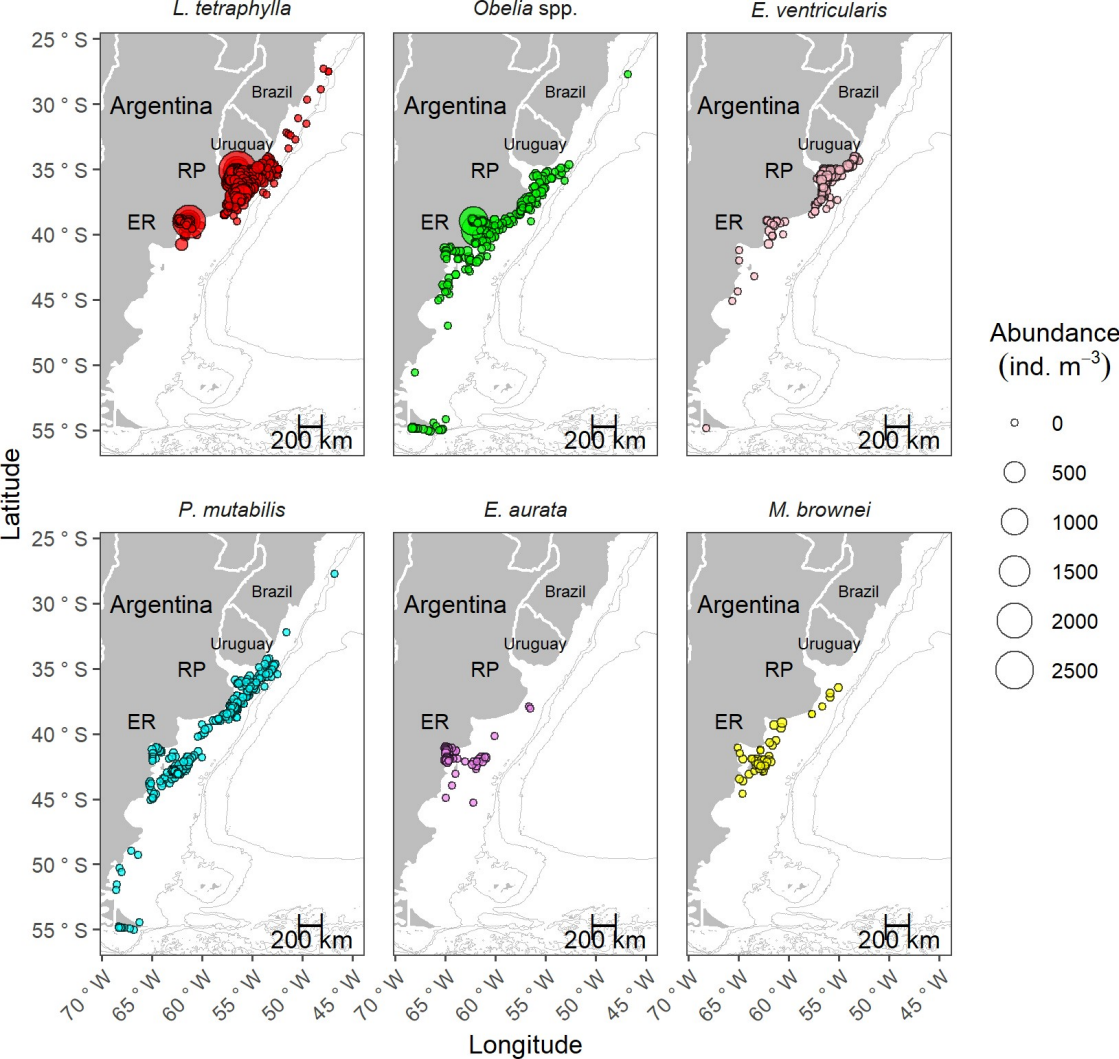
Spatial distribution of the abundance (ind. m-3) of the six most abundant hydromedusan taxa/species analyzed (in descending order of abundance: Liriope tetraphylla, Obelia spp., Eucheilota ventricularis, roboscydactila mutabilis, Euphysa aurata, and Mitrocomella brownei) along the SWA (27°-56°S) from 1983 to 2014. Data is shown in a continuous scale (min. value = 0.0017 ind. m-3, max. value = 2,474.49 ind. m-3). RP: Río de la Plata, ER: El Rincón.
